# Local cell metrics: a novel method for analysis of cell-cell interactions

**DOI:** 10.1186/1471-2105-10-350

**Published:** 2009-10-23

**Authors:** Jing Su, Pedro J Zapata, Chien-Chiang Chen, J Carson Meredith

**Affiliations:** 1 Coulter School of Biomedical Engineering, Georgia Institute of Technology, Atlanta, GA 30332, USA; 2 School of Chemical & Biomolecular Engineering, Georgia Institute of Technology, Atlanta, GA 30332, USA

## Abstract

**Background:**

The regulation of many cell functions is inherently linked to cell-cell contact interactions. However, effects of contact interactions among adherent cells can be difficult to detect with global summary statistics due to the localized nature and noise inherent to cell-cell interactions. The lack of informatics approaches specific for detecting cell-cell interactions is a limitation in the analysis of large sets of cell image data, including traditional and combinatorial or high-throughput studies. Here we introduce a novel histogram-based data analysis strategy, termed local cell metrics (LCMs), which addresses this shortcoming.

**Results:**

The new LCM method is demonstrated via a study of contact inhibition of proliferation of MC3T3-E1 osteoblasts. We describe how LCMs can be used to quantify the local environment of cells and how LCMs are decomposed mathematically into metrics specific to each cell type in a culture, e.g., differently-labelled cells in fluorescence imaging. Using this approach, a quantitative, probabilistic description of the contact inhibition effects in MC3T3-E1 cultures has been achieved. We also show how LCMs are related to the naïve Bayes model. Namely, LCMs are Bayes class-conditional probability functions, suggesting their use for data mining and classification.

**Conclusion:**

LCMs are successful in robust detection of cell contact inhibition in situations where conventional global statistics fail to do so. The noise due to the random features of cell behavior was suppressed significantly as a result of the focus on local distances, providing sensitive detection of cell-cell contact effects. The methodology can be extended to any quantifiable feature that can be obtained from imaging of cell cultures or tissue samples, including optical, fluorescent, and confocal microscopy. This approach may prove useful in interpreting culture and histological data in fields where cell-cell interactions play a critical role in determining cell fate, e.g., cancer, developmental biology, and tissue regeneration.

## Background

Cell-cell recognition is critical to a wide range of problems in biology and medicine [1-16]. The development of experimental approaches associated with cell-cell recognition has promoted advances in understanding these effects, e.g., biochemical assays for protein binding and transcription,. However, less attention has been focused on developing algorithms for the detection of cell-cell recognition from the structure and spatial distribution of cells. Such methods would offer complimentary benefits to biochemical assays, due to the relative ease of collecting microscopy data from cell cultures and tissues. This would be useful also in combinatorial and high-throughput screening of cell-cell and cell-material interactions [17-23]. In adhesion dependent cells, cell-cell recognition is known to be a crucial step in initiating contact inhibition (CI) of cell migration[24,25] and proliferation[14]. CI plays an important role in the proliferation, invasion, and metastasis of cancer cells, [26-28] cardiovascular tissue homeostasis and development, [5,29] embryonic development, [1] and wound healing, [16] among many other biological phenomena. Conversely, it has also been shown that under certain conditions cell-cell contact can promote cell proliferation, known as "density-dependent" contact stimulation of cell proliferation[12,30-32]. The investigation of mechanisms relating cell contact, cell proliferation, migration, and differentiation, in which cell adhesion molecules play a major role, is a rich area of research. Cadherins, e.g., VE-cadherin, [3] E-cadherin[13] and N-cadherin, [8,9,15,33] and notch proteins[1] have been shown to mediate contact-dependent phenomena in a wide variety of cell types.

Here, CI of proliferation, a known cell-cell recognition phenomenon, is used as a model system for developing algorithms for the analysis of cell-cell recognition from microscopy data. Usually, the effects of cell density on proliferation are studied as relationships between *global descriptions*, such as average cell density, proliferation rate, and protein expression level[2,11,12,14]. However, as we demonstrate below, these summary-statistic descriptions are only sensitive to the effects of very large changes in cell density. As a result, global metrics do not illuminate all of the information available from image data for cell contact phenomena. This is because cell-cell contacts are *local interactions *and are very sensitive to short-range cell-to-cell distance. When global metrics are used, such as cell density in a set of images, all cell-to-cell distances are treated equally. Critical information pertaining to nearest-neighbor cell-to-cell distances is "diluted" by the many other cell-to-cell distances in the data set, which is observed as noise in the data. Furthermore, the stochastic characteristics of cell behavior add to the noise so that only major trends of the responses of cells to neighbors is distinguishable over very large changes in cell density[15]. To address this "dilution" effect, Nelson and Chen studied contact stimulation effects on the growth of a single pair of cells by using specially-designed surfaces to decouple the effects of cell-cell contact from others[12].

In this paper, we introduce a complementary approach that allows focused analysis on nearest-neighbor cells, but permits sampling from cultures with high cell densities and use of any type of surface. We had previously applied this technique to screen large image databases from cell cultures on combinatorial libraries of biomaterials[22,23]. Here, we outline the details of this method and discuss it's generalization to Bayesian modelling. The method is based upon defining local cell metrics (LCMs), which are histograms of cell properties. The use of these local variables expands the sample space considerably and allows separation of arbitrarily-defined short- and long-range effects. We show how the local cell metrics are then incorporated into a Bayesian model. The new method and model are examined quantitatively and compared with traditional summary approaches in a study of contact inhibition of osteoblast proliferation.

## Methods

### Experimental

#### Surface Preparation

Poly (DL-lactic-glycolic acid) (PLGA, block copolymer, 50:50 ratio of PGA and PLA, 40,000~75,000 Da) and poly (ε-caprolactone) (PCL, 114,000 Da, M_w_/M_n _= 1.43) were obtained from Sigma Aldrich, St Louis, MO. PLGA and PCL, respectively, were dissolved in chloroform to 8% and 5% mass and spin coated on silicon chips (22 × 22 mm). To provide adhesion of these polymers to the silicon during cell culture, the silicon was pretreated with a Piranha etch (70% H_2_SO_4_/21% H_2_O/9% H_2_O_2 _at 80°C for 1 h) followed by 1 min in a HF acid bath and a final rinse in DI water (filtered at 0.2 μm).

#### Cell Culture

Established from newborn mouse calvaria, [34] the MC3T3-E1 cell line has been shown capable of differentiating into osteoblast and osteocytes *in vitro*[35]. MC3T3-E1 cells have been shown to exhibit specific bone related protein expression patterns, under different developmental stages, similar to primary mouse calvaria cells[35,36]. This cell line is thus a suitable *in vitro *model for investigating cell behaviors, regulations of such behaviors, and underlying mechanisms in different osteoblast maturation stages[37]. Since the original MC3T3-E1 cell line has been found phenotypically heterogeneous with regard to cell differentiation, more homogeneous subclones have been established[38]. In this work, MC3T3-E1 subclone 4 (from ATCC, VA), which shows homogenous capabilities of osteogenesis both *in vitro *and *in vivo*, [38] was chosen in order to minimize variations due to phenotypical heterogeneities.

Cell proliferation was assayed by BrdU immunohistochemistry. Briefly, PLGA- and PCL-coated wafers were mounted into Costar^® ^6-Well TC-Treated Microplates (Corning, NY). The tissue culture treated polystyrene (TCPS) surfaces of the microplate wells were used as controls. After sterilization (70% ethanol solution, 30 min), MC3T3-E1 cells (passage 6) were seeded onto the coated wafers at 4 × 10^4 ^cells/cm^2^. This relatively high seeding density was selected to highlight the effects of contact inhibition of cell growth and other space-sensitive cell-to-cell interactions. After seeding, microplates were shaken for 10 min on a shaker (Instrument model, operation frequency) to obtain uniform seeding. Cells were cultured in DMEM (Cellgro^® ^Dulbecco's Modification of Eagle's Medium, Mediatech, Inc., VA) with 10% fetal bovine serum (ATCC^® ^SCRC-1002™, ATCC, VA), L-glutamine and streptomycin at 37°C in a humidified 5% CO_2 _atmosphere. At 5 h post seeding, surfaces were washed with Dulbecco's Phosphate-Buffered Saline (DPBS, with Ca^++ ^and Mg^++^) to remove non-attached cells, and fresh culture medium was then added. At 18 h post seeding, 2 mM BrdU (5-bromo-2-φ-deoxyuridine, Sigma, MO) in PBS was added to the culture medium to reach a final concentration of 20 μM. After 6 h of BrdU incorporation, cells were fixed with 3.6% paraformaldehyde and BrdU incorporation was assayed by immunohistochemistry (primary antibody: mouse anti-BrdU, BD Biosciences, CA; secondary antibody: goat anti-mouse, Rhodamine conjugated, Rockland Immunochemicals, Inc., PA; counter staining: Hoechst 33342, Molecular Probes, Invitrogen Corporation, CA).

Low calcium concentration suppresses contact inhibition of cell growth by deactivating calcium-dependent cadherins[7,39]. This phenomenon was used in this study to validate the local cell metrics, and at the same time the dependency of contact inhibition on calcium was quantitatively studied. In order to investigate the role of Ca^++ ^on cell spreading and proliferation, BrdU incorporation experiments in low Ca^++ ^medium were performed on TCPS surfaces. Fifteen minutes before the introduction of BrdU, cells were rinsed twice with DPBS (without Ca^++ ^and Mg^++^) and afterword cultured in the low Ca^++ ^medium (0.5% FBS in Ca^++ ^and Mg^++ ^free DPBS)[39]. The rest of the protocol was the same as previously described.

#### Image Acquisition

Cell locations and proliferation were quantified using fluorescent microscopy (Olympus BX51 Clinical Microscope). A robotic translation stage was used to image predetermined locations on each culture surface using a MicroFire™ monochromic digital camera (SKU S99826, Optronics, CA). The image locations were fixed on a 16 × 20 grid with horizontal and vertical spacing of 1280 μm and vertical spacing of 960 μm. For each location a 1189 × 892 μm^2 ^BrdU staining image and Hoechst counter staining image were acquired at a resolution of 1600 × 1200 pixels^2^. All images and contextual information were organized and stored in an Oracle^® ^10 g (Oracle, CA) database for further image processing and data analysis.

#### Image Processing

The Image Processing Toolbox of Matlab™ R14 (MathWorks, MA) was employed for image processing. Due to the volume of image data dynamic, self-adapting algorithms were developed for automated image processing. Binary images of both surface lateral patterns of cell nuclei counter staining were obtained from raw grayscale microscopic images by a *variation-adjusted iterative selection method *(*VAIS*), which was modified from the original iterative selection method [40-45] (details in the BrdU thresholding part below).

Binary images of cell nuclei were segmented by the *marker-controlled watershed *method[42] to separate images of closely-spaced cell nuclei. This process was critical because the nuclei of a pair of recently-proliferated cells were often too close to be distinguished with thresholding alone. Resultant black-and-white cell nuclei images were used as masks by overlaying them with corresponding BrdU staining images to determine the fluorescence intensity of incorporated BrdU. The histogram of BrdU staining intensity per nucleus [see Additional File 1: Figure S.1] is composed of two major peaks: the low intensity peak (background) represents cells at rest, while the high intensity peak (foreground) indicates proliferating cells. The optimal threshold between these two peaks was determined automatically by *VAIS*. Briefly, starting at an initial threshold *T*_*i *_= 0.5, the histogram was divided into resting (background) and proliferating (foreground) parts. Means and standard deviations of the foreground and background, respectively denoted as *M*_*bi*_, *M*_*fi*_, *σ*_*bi*_, and *σ*_*fi*_, were determined by fitting each peak to a Gaussian curve. A new threshold was calculated as *T*_*i*+1 _= (*σ*_*fi*_*M*_*fi *_+ *σ*_*bi*_*M*_*bi*_)/(*σ*_*fi *_+ *σ*_*bi*_) and was repeated until convergence on a stable threshold. Compared with more common iterative selection methods, which use a simple mean intensity, the modified VAIS procedure is more robust when background and foreground intensities have different variances. Indeed, the variance of the BrdU signal intensity from non-proliferating cells was significantly greater than that of the proliferating cells [see Additional File 1: Figure S.1]. During image processing, data washing in the form of median filtering was performed to remove noise below a certain threshold. Image processing was supervised in order to assure the performance of self-adaptive algorithms and images of poor quality not permitting quantification were occasionally discarded. Proliferation behaviors were determined for every cell and stored in the database along with the cell location on the surfaces.

### Methodologies of Data Analysis

#### Global Metrics

**Figure 1:**
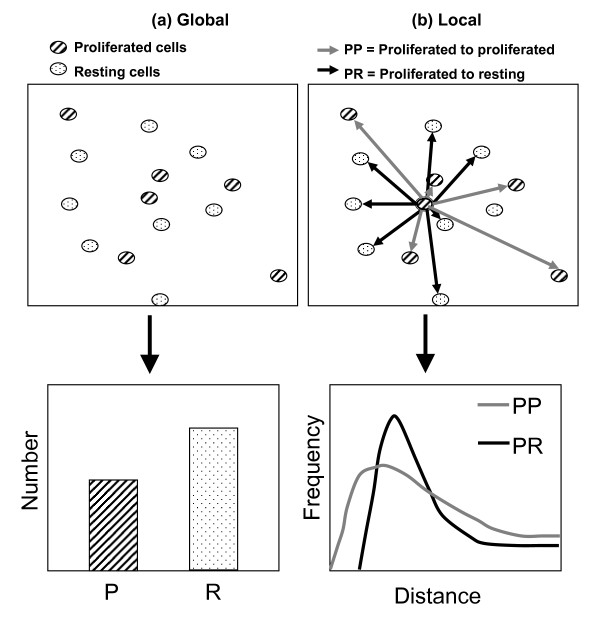
**Schematic comparing global versus local metrics**. Schematic comparing global versus local metrics. (a) global point of view leading to summary statistics, illustrated by bar graph, (b) local or individual-cell point of view leading to histograms, illustrated by frequency plot.

Cell density and proliferation were described with summary statistics such as number of resting and proliferated cells computed for each image. This provides a set of global metrics for features in each image. As indicated in Figure 1a, global metrics are most naturally understood in terms of conventional summary-statistics, exploratory data analysis, and well-known methods for estimating confidence and significance levels based on an assumed probability distribution. The ability to detect contact inhibition of cell proliferation, a known phenomenon, was used as an indicator of the effectiveness of the global metrics cell density and proliferation averages.

#### Definition of Local Cell Metrics

Source codes that implement the algorithms presented in this section have been made available by the authors. [see Additional File 2] Consider that the collection of all cells (A) is composed of either proliferated (P) or resting cells (R), such that A = P + R. The symbol A represents any cell chosen at random, regardless of proliferative status. The proliferating-resting cell distance, *PR*, is used here to illustrate the definition and properties of local cell metrics, as indicated in Figure 1b. The definitions below are generalizable to any type of cell-cell distance, or any other spatial or temporal metric of cells. Assume that in the *k*^th ^image the number of P-class and R-class cells is *n*_*Pk *_and *n*_*Rk*_, the distance *PR*_*ijk *_between the centroids of the nuclei of the *i*^th ^P-cell and the *j*^th ^R-cell can be calculated readily from the results of image analysis. In the *k*^th ^image, the set of all such distances, *PR*_*k *_is defined as(1)

And for all images an overall set *PR *can be defined as(2)

A set of *N*+1 distance bins is defined as(3)

where *d*_0_, *d*_1_, ..., *d*_*N *_is a user-defined distance scale over which analysis is to be performed. The centroid of each interval in *bin*_*dist *_is defined as(4)

and the resultant centroid set for *bin*_*dist *_is(5)

The *bin*_*dist *_is used to sort set *PR *into an *N*-bin histogram(6)

where  is the number of *PR *distances that fall in the interval [*d*_*i*-1_, *d*_*i*_), which is centered at .

The total number of elements in set *PR *is(7)

After normalizing by *n*_*PR*_, the frequency function LCM is(8)

and  represents the R-type cell environment of the P-type cells observed over distances . Frequency functions, denoted as , , , and , may also calculated for cell-to-cell distances *PP*, *AA*, *RR*, and *PA *in similar manner.

#### LCM Normalization

Normalization is necessary to interpret LCMs in a meaningful manner and to compare the probability of cell responses under different cell environments. One method of normalization is to relate observed occurrences to random occurrences. Given the finite image size and generally non-overlapping nature of cultured cells, the distribution of random cell occurrences is not Gaussian. The random distribution for cell-cell distance, *f*_*std*_, was calculated as the any cell-any cell distribution (*f*_*AA*_) of 1× 10^10 ^randomly-chosen nuclei positions on a simulated image 1600 pixels by 1200 pixels. The normalized LCM  is(9)

Other LCMs (*f*_*AA*_, *f*_*PA*_, *f*_*RR*_) are normalized similarly, which allows direct comparisons of different types of cell distances on different surfaces.

In addition to normalizing by the standard distribution, *f*_*std*_, direct ratios between LCMs are used also in our analysis, in which case *f*_*std *_cancels, as indicated in the next equation.(10)

The ratio *r*_*PR*|*PA *_highlights the specific effects of non-proliferated cells on the central proliferating cell relative to the effects of any given cell. Thus, the probability of cell responses under different cell environments can be compared meaningfully. Furthermore, each set of cell-to-cell distances can be decomposed into subsets, which allows investigation of the contribution of each subset to the overall effect. Therefore, ratios of cell backgrounds may be constructed and used as classifiers for screening and identifying significant cell environment patterns. These ratios also define *posterior odds *(PO) of observing certain proliferation behaviors. For example, consider , and using the subscript *i *to signify the evaluation at a certain distance , the ratio is calculated as(11)

where(12)(13)

Applying equations (12) and (13) to equation (11),(14)

Thus,  is a posterior odds that quantifies how the probability of cell proliferation is changed by the presence of a second cell located at distance , relative to the average proliferation for all cell-cell distances. Computationally, to promote the efficiency of the codes, we defined *A*_*k *_= {*P*_*k*_, *R*_*k*_}={*P*_1*k*_, *P*_2*k*_, ..., *P*_*NPk*_, *R*_1*k*_, *R*_2*k*_, ..., *R*_*NRk*_}, and removed self-to-self cell pairs (*PA*_*ijk *_where *i = j*) and identical cell pairs (*PA*_*ijk *_where *i *>*j*) from *PA*.

#### LCM Decomposition

**Figure 2:**
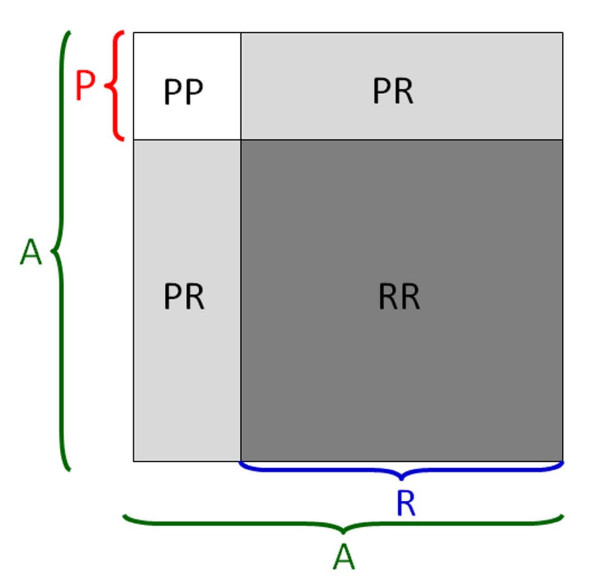
**Schematic indicating the decomposition of local metrics**. Schematic indicating the decomposition of local metrics into groups of cell-cell distances based on cell proliferative status. A = any cell, P = proliferated cell, R = resting cell (nonproliferated).

Furthermore, each set of cell-to-cell distances can be decomposed into subsets, which allows isolation of each subset's contribution. For example, consider *r*_*PR*|*PA *_defined above. As described graphically in Figure 2, since the denominator *PA *= *PP *∪ *PR *(the union of distance sets *PP *and *PR*) and *PP *∩ *PR *= ∅ (∅ = the empty set), one may remove the *PR *component from *PA*, and the denominator becomes *PP*. The result is that the ratio *r*_*PR*|*PA *_is transformed into *r*_*PR*|*PP*_. By removing the shared, or overlapping, component *PR *from the denominator, *r*_*PR*|*PP *_has higher "contrast" for observing effects of R-cells on P-cells than *r*_*PR*|*PA*_.

#### Connection of LCMs to Bayesian Statistics

Local cell metrics are naturally connected to Bayesian analysis, which is a powerful statistical method used for classification[46,47]. Specifically for the *PR *distance, the Bayesian approach allows one to quantify the local environment of P cells, as the conditional probability of finding an R cell a certain distance *PR *from a P cell. Based on the definition of *f*_*PR *_in equation (8), a naïve Bayes model can be established as follows. Consider a "test" cell chosen at random. It is desired to predict the possibility this cell will be in proliferating status, based upon the local environment of non-proliferating cells, which is given by the following conditional probability function(15)

where  represents the probability of finding a non-proliferating cell at a distance of  from the central, randomly chosen, test cell. Using Bayes's theorem,(16)

In the above function, the components *p*() and *p*(*prolif*) are constants that can be determined from their frequency in the data. The only non-constant component, the class-conditional probability, is given by(17)

Assuming the occurrence probabilities around the non-proliferating cell distances  are conditionally independent (uncorrelated), then  where *i *≠ *j *(the naïve Bayes assumption). Hence, equation (17) reduces to(18)

A key development is to notice that , which means that under the naïve Bayes assumption the LCM is in fact a class-conditional probability function. The term *p*() represents the probability of locating the R cells at distances () from any cell, which is . Hence, the Bayes conditional probability function from equation (16) becomes(19)

The naïve Bayes model allows prediction of the probability of proliferation as a function of the LCMs,  and , which are easily computed from a training data set, as is *p*(*prolif*). The evaluation of this modeling approach will be the subject of forthcoming work.

## Results and Discussion

### Traditional metrics

**Figure 3:**
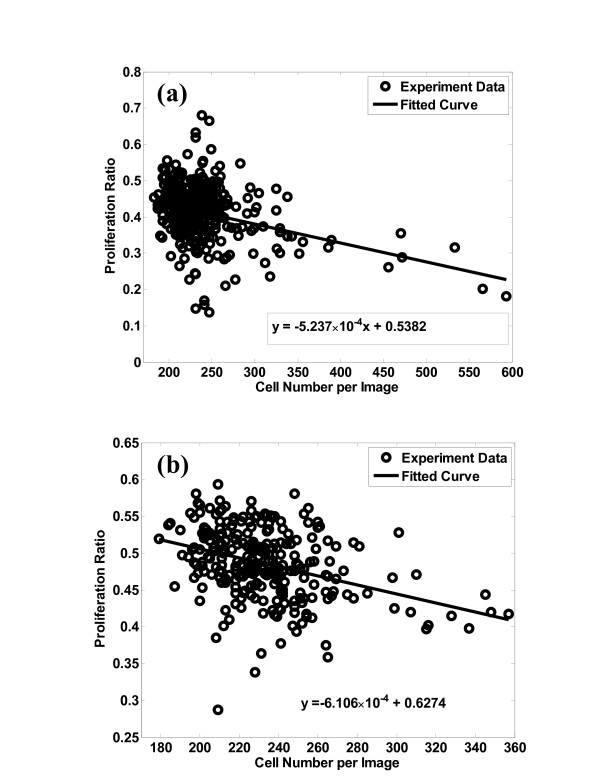
**Effects of global cell density on global cell proliferation**. Effects of global cell density on global cell proliferation ratio for MC3T3-E1 cultured on (a) PLGA and (b) PCL. Number of images used was (a) 353 and (b) 288.

To provide a benchmark for establishing the effectiveness of local metrics, contact inhibition of cell proliferation was studied using global analysis first. For each image in the database, the overall cell proliferation is plotted versus cell density, shown in Figure 3 and Table 1. Although a trend towards lower proliferation at higher cell density is somewhat apparent in Figure 3, global analysis, does not allow for quantitative detection of CI effects on cell proliferation at a statistically-significant level. The linear regression (Table 1) yields in an adjusted *R*^2 ^of 0.128 (on PLGA) or 0.109 (on PCL), indicating that the contact inhibition effect masked by "noise" in the data. Furthermore, it is obvious that in Figure 3, no regression function can be fit satisfactorily to the global statistics, since the noise is too high relative to the CI effects. The use of a larger range may allow the global analysis to distinguish contact effects from natural variance in cell properties. This has been demonstrated in repeated experiments on PLGA, PCL, and TCPS surfaces. [see Additional File 1: Figures S.2, S.3, S.4]. However, there are drawbacks to the use of larger ranges, such as the introduction of seeding-density effects that mask or alter the cell-cell interactions.

**Table 1:** Linear Regression from Global Analysis Results from Figure 3

Surface	**Coeff**.	SSE	R^2^	RMSE	Adj R^2^
PCL	-6.106 × 10^-4^	0.5314	0.1312	0.04396	0.1280
PLGA	-5.237 × 10^-4^	1.846	0.1111	0.07251	0.1086

*SSE = Sum of squared error, RMSE = root mean squared error, R^2 ^= linear correlation coefficients

### Local Cell Based Metrics

**Figure 4:**
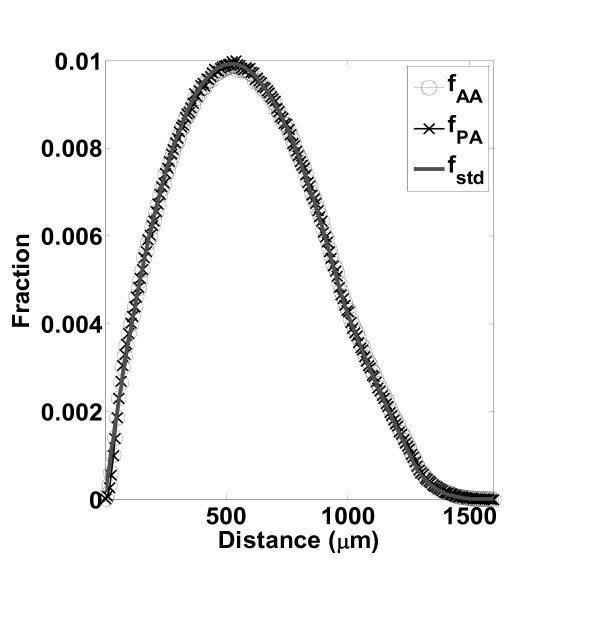
**Comparison of experimental and calculated distributions**. Comparison of experimentally determined *f*_*AA *_and *f*_*PA *_for MC3T3-E1 on a PLGA surface and the computed standard curve, *f*_*std*_. These data represent the frequency at which any cell is located a certain distance from any other cell (*f*_*AA*_) or from a proliferated cell (*f*_*PA*_) in the experiments. The standard curve was computed from a Monte Carlo simulation and represents a uniformly random probability of locating any cell a given distance from any other cell on the same sized area as our microscope images. Number of images used was 353.

The noise level inherent to proliferation measurements, which are normally carried out over a small seeding density range, make contact inhibition a robust test-case for comparing local *vs*. global metrics. A contact phenomenon is detected when a relevant metric changes significantly relative to the data sampling noise. For global statistics, the assumed distribution (usually Normal) provides the random noise reference. For local metrics, the random cell-cell distance frequency distribution was calculated using a Monte-Carlo approach, termed the standard frequency distribution, *f*_*std*_. The reference *f*_*std *_is shown in Figure 4 together with the experimental *f*_*AA *_for MC3T3-E1 osteoblasts on PLGA. The profile of *f*_*std *_is similar to a beta- or chi-distribution with asymmetry due to the non-overlapping nature of the nuclei centers at close distances. The computed *f*_*std *_distribution is nearly identical to the experimental *f*_*AA *_distribution at large distances (> 100 μm). This is expected since *f*_*AA *_indicates the likelihood of finding any two cells (whether proliferating or not) separated by a given distance, which should in principle be random. Figure 4 also shows the distance distribution *f*_*PA*_, which is the likelihood of finding a proliferated cell a certain distance from any cell. If cell-cell distance has any relation to proliferative status then *f*_*PA *_and *f*_*AA *_should differ from one another and from *f*_*std*_, but only at close distances where cell-cell contact is likely to occur.

**Figure 5:**
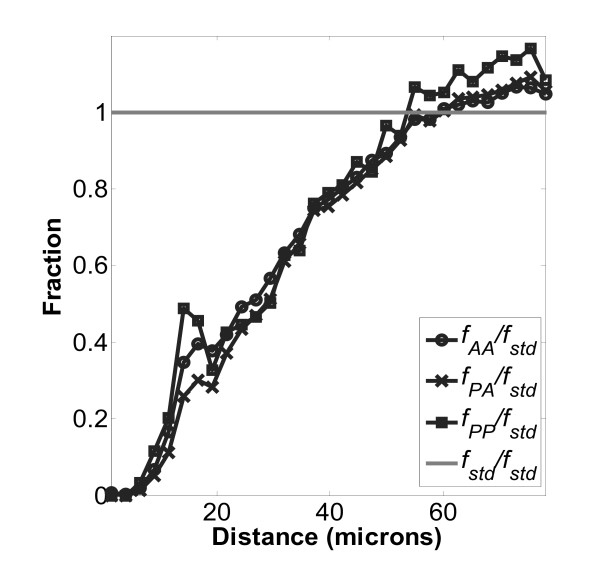
**Normalized distributions for cells on PLGA surfaces**. Normalized distributions for any cell-any cell (AA), proliferating-any cell (PA) and proliferating-proliferating cell (PP) on a PLGA surface: , , and . Normalization was performed by dividing the experimental frequency distribution by *f*_*std*_, the random cell-cell distribution determined from Monte Carlo simulation. Number of images used was 353.

**Figure 6:**
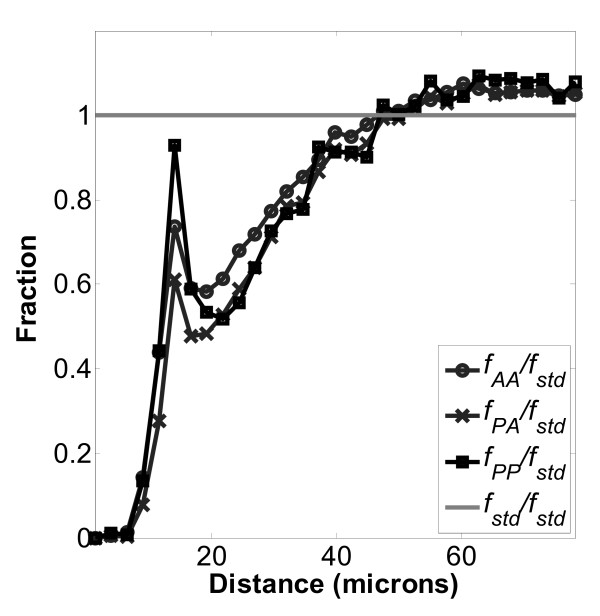
**Normalized distributions for cells on PCL surfaces**. Normalized distributions as described in Figure 5 on a PCL surface: , , and . Number of images used was 288.

**Figure 7:**
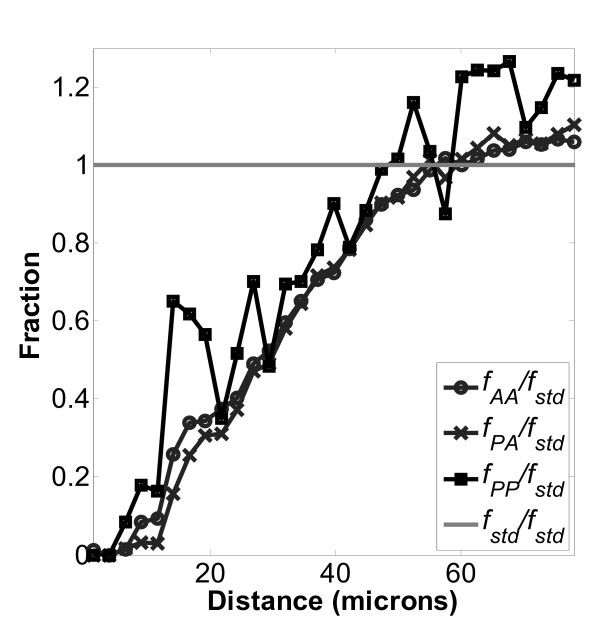
**Normalized distributions for cells on TCPS surfaces**. Normalized distributions as described in Figure 5 on a TCPS surface: , , and . Number of images used was 291.

**Figure 8:**
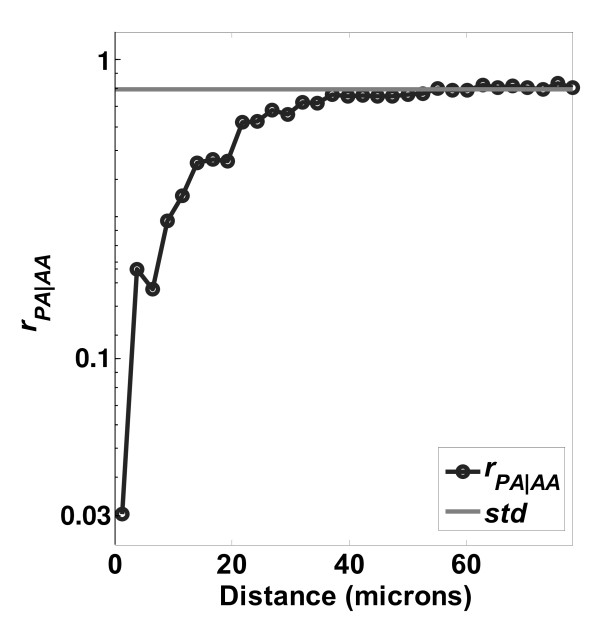
**Ratio of distributions PA/RR on PLGA surfaces**. Ratio of distributions  on a PLGA surface. Number of images used was 353.

**Figure 9:**
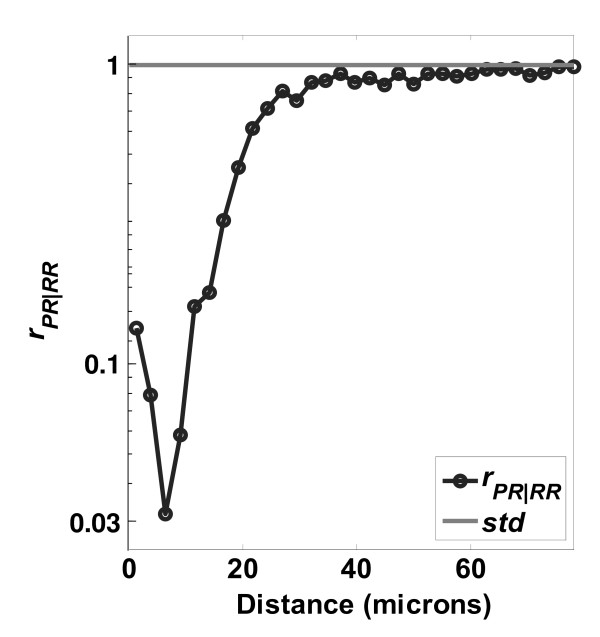
**Ratio of distributions PR/RR on PLGA surfaces**. Ratio of distributions  on a PLGA Surface. Number of images used was 353.

**Figure 10:**
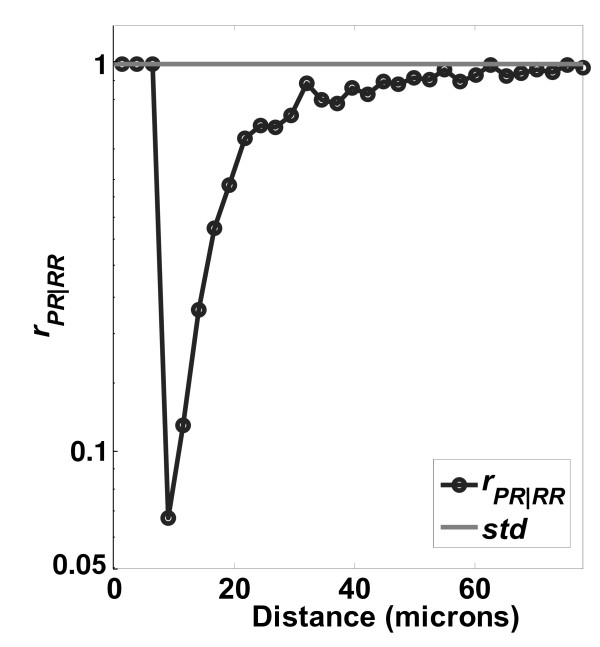
**Ratio of distributions PR/RR on PCL surfaces**. Ratio of distributions *r*_*PR*|*RR *_on a PCL surface. Number of images used was 288.

**Figure 11:**
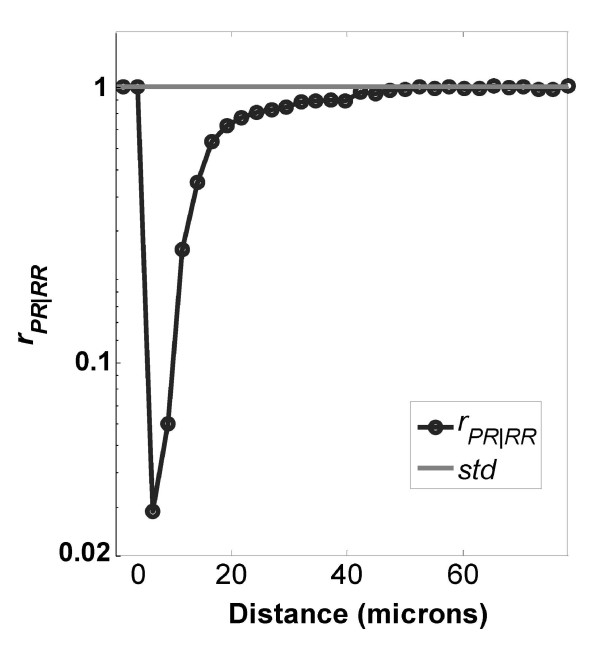
**Ratio of distributions PR/RR on TCPS surfaces**. Ratio of distributions *r*_*PR*|*RR *_on a TCPS surface. Number of images used was 291.

Figures 5, 6, and 7, which show the normalized distributions *f*_*PA*_/*f*_*std*_, *f*_*AA*_/*f*_*std *_and *f*_*PP*_/*f*_*std *_at close distances, indicate the non-random effects of contact inhibition when the values become less than one. Specifically, CI occurs when the distance between cell nuclei becomes less than about 50 μm. The typical mean cell area was around 2500 μm^2^, resulting in a mean diameter of 56 μm, which corresponds closely to the onset of CI. Representative images of cultured MC3T3-E1 cells on these surfaces have been presented in previous work[22,23]. In addition, local fine structure in the contact inhibition region is observed as a local maximum peak between 10 and 20 μm. This peak indicates enhanced local proliferation at very close distance, even when overall proliferation is being inhibited. Interestingly the local peak magnitudes at 10 to 20 μm always follow the order *f*_*PP *_>*f*_*AA *_>*f*_*PA *_on each of the three surfaces examined, TCPS, PLGA and PCL. We hypothesize that the local enhancement peak is due to two daughter cells (from the same parent cell) that are very close, which have not had enough time to migrate away during the BrdU staining time period. If so, then this cell division peak should appear on the *f*_*PP *_curve but not the *f*_*PR *_curve, which was observed comparing Figures 5, 6, and 7 (*f*_*PP*_) to Figures 8, 9, 10, and 11 (*f*_*PR*_). In addition, in the Monte Carlo simulation of random cell positions (*f*_*std*_), with no proliferation, this local peak is absent.)

Direct ratios between experimentally-determined distributions can be chosen specifically to illuminate the CI phenomena of interest. Specifically, common components in the numerator and denominator not related to CI phenomena may be removed, thus isolating the phenomena of interest. This process is examined in Figures 8, 9, 10, and 11. Figure 8 shows the *r*_*PA*|*AA *_profile, which is classified into two regions: the *proliferation suppressed region *(0 to 40 μm) where cell proliferation was suppressed up to 6 fold (*r*_*PA*|*AA *_falls to ~1/6) and the *null region *(beyond 40 μm) where cell proliferation was not noticeably affected by the contact of other cells. Based on the discussion for Figure 7, the ratio *r*_*PA*|*AA *_does not fully decouple the division and daughter-cell migration phenomena (indicated by PP) from the proliferation phenomena (indicated by *PR*), since *PP *⊂ *PA*. We illustrate how separation of the *PP *and *PR *components enhances the detection of contact inhibition of proliferation. By definition the various distances are related as follows(20)

with(21)

where ∅ is the empty set. The two shared components of *AA *and *PA *are *PP *and *PR*. The *PP *distance component represents distances between cells that have both proliferated, i.e., *proliferation in those pairs was not contact inhibited*. Removal of the common *PP *component from the numerator and denominator of *r*_*PA*|*AA *_leads to *r*_*PR*|*RR*_, shown in Figures 9, 10, and 11 for the PLGA, PCL, and TCPS surfaces, respectively. By definition *r*_*PR*|*RR *_should be more sensitive to CI of proliferation, because non contact inhibited cell pairs have been removed. In Figure 9, the *r*_*PR*|*RR *_ratio is classified into two regions: the *NaN region *(below 5 μm) where few pairs occur, and the *contact inhibition region *(5 to 40 μm). In the *contact inhibition region*, a clear trend of decreasing probability of finding a neighboring cell is seen as the distance between cells decreases. A minimum is observed at *d*_*min *_= 8 μm, where contact inhibition effects are maximized. To our knowledge, this is the *first time both the magnitude and the range of contact inhibition of cell proliferation *have been determined quantitatively in a single function.

The physical meaning of the LCM ratio *r*_*PR*|*RR *_can be can be illustrated by recognizing that it is the posterior odds (PO) of proliferation as a function of cell-cell distance. Consider two cells that are well-separated at 40 μm, and another two cells that are at a close distance of 8 μm, where the extreme in contact inhibition behavior is found (minimum *r*_*PR*|*RR *_in Figure 9). The PO that one of the closely-spaced cells has proliferated is *PO*_*PR*/*RR *_= *r*_*PR*|*RR *_(8)/*r*_*PR*|*RR *_(40) = 1/32. This means there is a 32 fold lower chance of proliferation at 8 μm than at a distance of 40 μm.

The profiles of *r*_*PR*/*RR *_from the other polymer surfaces are shown in Figure 10 (PCL surface) and Figure 11 (TCPS surface). The ratios are similar in shape but have different magnitudes for the minimum point as a function of the surface. Table 2 summarizes the variation of *r*_*PR*|*RR*, *min *_and *d*_*min *_on the different surfaces. The different location and strength of contact inhibition might be due to surface features such as roughness, crystallinity, hydrophobicity, surface charge, or protein adsorption, factors which are known to influence osteoblast proliferation[48]. For example, the surface roughness increases in the order TCPS < PLGA < PCL, and at the same time the *PO*_*PP*/*PR *_is decreasing, and the *d*_*min *_is increasing. We illustrate this point, however, not to make a definite mechanistic argument about surface effects on proliferation, which is certainly more complicated than roughness alone. Rather, the point made is that the LCM method is capable of sensitive detection of differences in proliferation for cells cultured on different surfaces.

**Table 2:** Minima in *r*_*PR*|*RR *_Curve Indicating Maximum Contact Inhibition

Surface	*1/r*_*PR*|*RR*, *min*_	*d*_*min*_(μm)
PLGA	31.6	8
PCL	15.8	9
TCPS	35.5	6

**Figure 12:**
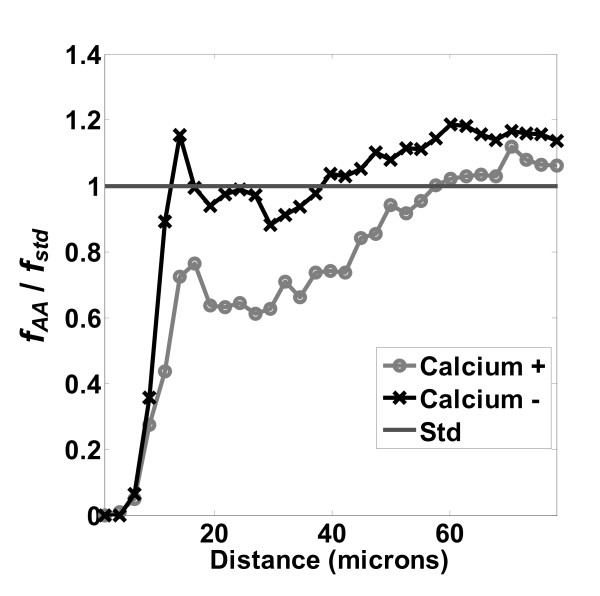
**Effects of calcium depletion: loss of contact inhibition**. Normalized distribution  examining effects of calcium depletion on proliferation of MC3T3-E1 cultured on TCPS surfaces. Number of images used was 198.

**Figure 13:**
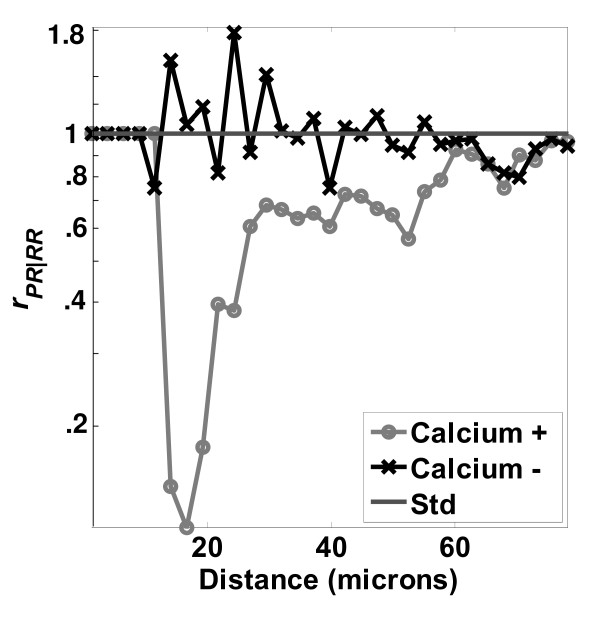
**Effects of calcium depletion: loss of contact inhibition**. Ratio of distributions *r*_*PR*|*RR *_examining effects of calcium depletion on proliferation of MC3T3-E1 cultured on TCPS surfaces. Number of images used was 204.

The effect of calcium depletion on LCMs is presented in Figure 12. The  distribution of the control (calcium +) was significantly lower than the low calcium (calcium -) case. In addition, the 'calcium -' curve stays close to unity except at very close distances, less than 20 μm, whereas the 'calcium +' curve falls below unity at 50 μm. Hence, the LCM  detects the expected result: that low calcium should inhibit the cell-cell self-avoidance and contact inhibition[7,39]. This effect is seen more clearly in examining the ratio *r*_*PR*/*RR*_, in Figure 13. Contact inhibition was very strong within a cell-to-cell distance of 30 μm when cells were cultured under physiological calcium concentration (calcium +). However, contact inhibition disappeared when calcium was depleted (calcium -).

## Conclusion

We have shown that global summary statistics are not adequate metrics for detecting local cell interactions, due to noise and non-local effects inherent to cell-cell contact phenomena. A novel data analysis strategy, *local cell metrics*, has been introduced in this paper. LCMs, which are cell-cell distance histograms, describe cell environments from the "point of view" of individual cells. These metrics allow focusing of analysis onto arbitrarily-defined close distances. In addition, LCMs can be 'tuned' to be sensitive to specific contact phenomena by decomposing the distributions into specific cell-types (proliferating vs. nonproliferating) and removing unwanted components. Local metrics as defined herein are not limited to proliferation analysis, nor to cell-cell interactions alone. The metrics are generic and can be, in principle, applied to any type of quantifiable cell assay, and can be applied to cell-biomaterial and cell-tissue interactions as well. We have also shown how LCMs are related to the naïve Bayes model, which makes them useful for data mining and classification (the subject of forthcoming work.)

We have demonstrated the new local metrics by considering the contact inhibition of proliferation of the osteoblast cell line MC3T3-E1. A quantitative and probabilistic description of the contact inhibition effect as a function of cell-cell distance has been achieved. In fact, the probability of proliferation is shown to be strongly dependent on the distance to, and proliferative state of, neighboring cells. The LCMs were also sensitive to effects of the culture surface, and of calcium composition in the culture media, on proliferation.

## Authors' contributions

JCM directed the experimental design, data analysis method development, and writing of the manuscript. JS performed most cell culture experiments and implemented the LCM method, including programming, and wrote the manuscript. PJZ helped in data interpretation and statistical analysis. CCC performed the calcium-depletion experiments and analysis. All authors have read and approved this manuscript.

## Supplementary Material

Additional file 1 **Supplementary materials**. Supplementary theoretical and calculation details, as well as additional data on image analysis and global analysis are provided. Click here for file

Additional file 2 **Source codes for local cell metrics**. This .zip archive folder contains sources codes for implementing local cell metrics provided under terms of the GNU open source license. Click here for file
