# Supplementary material for: Local cell metrics: a novel method for analysis of cell-cell interactions
Source: BMC Bioinformatics. 2009 Oct 23;10:350. doi: 10.1186/1471-2105-10-350 (PMC2944256; doi:10.1186/1471-2105-10-350)
Supplement: Additional file 1 — Supplementary materials. Supplementary theoretical and calculation details, as well as additional data on image analysis and global analysis are provided. [file 1471-2105-10-350-S1.DOC]

**Supplementary MATERIALS**

**Theory**

**Source Codes are available as supplementary material from the publisher and at the project website at** [**www.bioinformatics.org/lcmets**](http://www.bioinformatics.org/lcmets)**.**

We did not explicitly notate the difference between the mathematical expectation of the variable, *E(V)*, and the variable itself, *V*, as long as the assumptions are valid that the specific variable follows normal distribution and the sample size is large.

Strictly, assuming normal distributions of
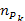
 and
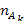
, since
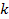
 is large (
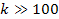
),


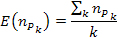
 (S.1)

and


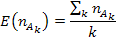
. (S.2)

Equation (12) would be:


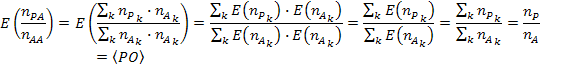
 (S.3)

Equation (13) wuld also be strictly expressed as:


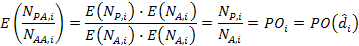
 (S.4)

Since it is common to omit the notations of mathematical expectations as long as the variables follow normal distribution and the sample size is large, we hereby provide both forms and suggest using the simplified forms in text while referring readers to the strict forms in the supplemental material.

**Extension to other distances.** Manuscript equations 1, 2, 6, 7, and 8 use set PR as an example for the normalization method that is also used for each other set. As stated in the manuscript on page 10, “Frequency functions, denoted as
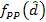
,
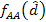
,
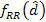
, and
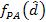
, may also calculated for cell-to-cell distances *PP*, *AA*, *RR*, and *PA* in similar manner.” The equations of other sets are similar as those of set PR. Below, we use set
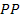
 as an example to show the definitions corresponding to Equation 1, 2, 6, 7, and 8.


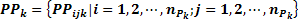
 (S.5)


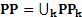
 (S.6)


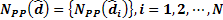
 (S.7)


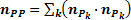
 (S.8)


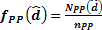
 (S.9)

Generally speaking, when
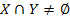
 (e.g., for set
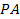
,
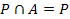
, and for set
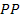
,
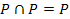
), the overlapped elements in
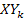
 will represent the distance from one cell to itself. The effects of such self-to-self distance are normalized in Equation 8 (full text) and S.9 above, and thus cause no artifacts.

**Computational Details**

Computationally, to promote the efficiency of the codes, we defined
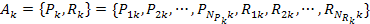
, and removed self-to-self cell pairs (
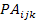
 where
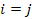
, that is, the self-to-self cell pair from
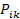
 to
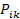
) and identical cell pairs (
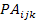
 where
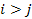
, that is, this cell pair is identical to
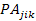
) from
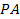
. Therefore,


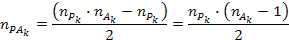
 (S.5)

and


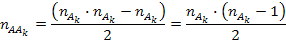
 (S.6)

and


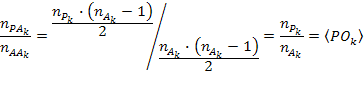
 (S.7)

**Supplemental Figures**

**Figure S.1**

Typical histogram from a single micrograph image of BrdU staining intensity, illustrating the automated selection of threshold for counting proliferated cells.

# Figure S.2

Global summary statistics: proliferation rate versus cell density from repeated experiments on PLGA surfaces.

# Figure S.3

Global summary statistics: proliferation rate versus cell density from repeated experiments on PCL surfaces.

# Figure S.4

Global summary statistics: proliferation rate versus cell density from repeated experiments on TCPS control surface.
